# Impact of SARS-CoV-2 Infection on Long-Term Depression Symptoms among Veterans

**DOI:** 10.1007/s11606-024-08630-z

**Published:** 2024-04-16

**Authors:** Jason I. Chen, David Bui, Theodore J. Iwashyna, Troy A. Shahoumian, Alex Hickok, Megan Shepherd-Banigan, Eric J. Hawkins, Jennifer Naylor, Diana J. Govier, Thomas F. Osborne, Valerie A. Smith, C. Barrett Bowling, Edward J. Boyko, George N. Ioannou, Matthew L. Maciejewski, Ann M. O’Hare, Elizabeth M. Viglianti, Amy S.-B. Bohnert, Denise M. Hynes, for the V. A. HSRDSARS-C.O.V.-2 Observational Research Collaboratory (CORC)

**Affiliations:** 1 Center to Improve Veteran Involvement in Care (CIVIC), VA Portland Health Care System (HCS), Portland, OR USA; 2 Department of Psychiatry, Oregon Health & Science University, Portland, OR USA; 3 Departments of Medicine and Health Policy and Management, Johns Hopkins University, Baltimore, MD USA; 4 Center for Clinical Management Research, VA Ann Arbor HCS, Ann Arbor, MI USA; 5 VHA, Patient Care Services: Health Solutions, Washington, DC USA; 6 Center of Innovation to Accelerate Discovery and Practice Transformation (ADAPT), Durham VA HCS, Durham, NC USA; 7 Department of Population Health Sciences, Duke University School of Medicine, Durham, NC USA; 8 Center of Innovation for Veteran-Centered and Value-Driven Care, VA Puget Sound HCS, Seattle, WA USA; 9 Center of Excellence in Substance Addiction Treatment and Education, VA Puget Sound HCS, Seattle, WA USA; 10 Department of Psychiatry and Behavioral Sciences, University of Washington, Seattle, WA USA; 11 School of Medicine, Department of Psychiatry and Behavioral Sciences, Duke University, Durham, NC USA; 12 VISN 6 Mental Illness Research, Education and Clinical Center, Durham, NC USA; 13 Durham VA HCS, Durham, NC USA; 14 OHSU-Portland State University School of Public Health, Oregon Health & Science University, Portland, OR USA; 15 VA Palo Alto HCS, Palo Alto, CA USA; 16 Department of Radiology, Stanford University School of Medicine, Stanford, CA USA; 17 Department of Medicine, Duke University, Durham, NC USA; 18 Durham VA Geriatric Research Education and Clinical Center, Durham VA HCS, Durham, NC USA; 19 Seattle Epidemiologic Research Information Center, VA Puget Sound HCS, Seattle, WA USA; 20 Division of Gastroenterology, Department of Medicine, University of Washington, Seattle, WA USA; 21 Hospital and Specialty Medicine Service, VA Puget Sound HCS, Seattle, WA USA; 22 University of Washington, Seattle, WA USA; 23 Division of Pulmonary and Critical Care Medicine, Department of Internal Medicine, University of Michigan, Ann Arbor, MI USA; 24 Department of Psychiatry, University of Michigan Medical School, Ann Arbor, MI USA; 25 College of Public Health and Human Sciences, and Center for Quantitative Life Sciences, Oregon State University, Corvallis, OR USA; 26 School of Nursing, Oregon Health & Science University (OHSU), Portland, OR USA

## Abstract

**Background:**

Prior research demonstrates that SARS-COV-2 infection can be associated with a broad range of mental health outcomes including depression symptoms. Veterans, in particular, may be at elevated risk of increased depression following SARS-COV-2 infection given their high rates of pre-existing mental and physical health comorbidities. However, few studies have tried to isolate SARS-COV-2 infection associations with long term, patient-reported depression symptoms from other factors (e.g., physical health comorbidities, pandemic-related stress).

**Objective:**

To evaluate the association between SARS-COV-2 infection and subsequent depression symptoms among United States Military Veterans.

**Design:**

Survey-based non-randomized cohort study with matched comparators.

**Participants:**

A matched-dyadic sample from a larger, stratified random sample of participants with and without known to SARS-COV-2 infection were invited to participate in a survey evaluating mental health and wellness 18-months after their index infection date. Sampled participants were stratified by infection severity of the participant infected with SARS-COV-2 (hospitalized or not) and by month of index date. A total of 186 participants in each group agreed to participate in the survey and had sufficient data for inclusion in analyses. Those in the uninfected group who were later infected were excluded from analyses.

**Main Measures:**

Participants were administered the Patient Health Questionnaire-9 as part of a phone interview survey. Demographics, physical and mental health comorbidities were extracted from VHA administrative data.

**Key Results:**

Veterans infected with SARS-COV-2 had significantly higher depression symptoms scores compared with those uninfected. In particular, psychological symptoms (e.g., low mood, suicidal ideation) scores were elevated relative to the comparator group (M_*Infected*_ = 3.16, 95%CI: 2.5, 3.8; M_*Uninfected*_ = 1.96, 95%CI: 1.4, 2.5). Findings were similar regardless of history of depression.

**Conclusion:**

SARS-COV-2 infection was associated with more depression symptoms among Veterans at 18-months post-infection. Routine evaluation of depression symptoms over time following SARS-COV-2 infection is important to facilitate adequate assessment and treatment.

**Supplementary Information:**

The online version contains supplementary material available at 10.1007/s11606-024-08630-z.

## INTRODUCTION

Depression is a leading cause of disability and disease burden in the United States (U.S.) with a lifetime prevalence of up to 21% and widespread impact on social and emotional functioning.^1^ Past research has demonstrated medical conditions resulting in increased inflammation, such as autoimmune conditions, are associated with increased risk of depression.^2,3^ Known associations of SARS-CoV-2 infection with acute and chronic inflammatory responses have raised the question of whether there is increased depression risk post-infection.^4,5^ A recent systematic review finds some associations between SARS-COV-2 infection and depressive symptoms with significant heterogeneity between studies.^6^ This review also identifies a notable limitation of the extant literature regarding evaluating symptoms past one-year post-infection. U.S. Military Veterans, in particular, may be at elevated risk for increased depression following SARS-CoV-2 infection in light of their high prevalence of mental and physical health comorbidities, which may exacerbate negative, post-infection health.^7,8^ This high prevalence of physical and mental health comorbidities is particularly concerning as research among non-Veterans finds that past mental health history is associated with increased risk of depression following SARS-COV-2 infection.^9^

Past studies have found some associations between SARS-COV-2 infection and depression symptoms.^10–12^ However, these studies had notable limitations such as limited ability to account for potential confounding variables (e.g., pre-existing physical and mental health comorbidities), biases inherent to administrative medical record data (e.g., limited capture of mental health diagnoses), abbreviated follow-up periods (1 year or less), and lack of self-reported depressive symptoms. In this study, we robustly matched SARS-CoV-2 infected patients with contemporaneous, uninfected controls to focus on the effects of SARS-COV-2 infection on depression symptoms to help inform tailoring of treatment to patient needs. In addition, we explored associations between SARS-CoV-2 infection and depression symptom subdomains (i.e., psychological, physical) to differentiate symptoms commonly associated with long-term symptoms of SARS-CoV-2 infection (e.g., low energy) from mood disturbance (e.g., negative self-worth).

In light of the need to better understand the unique association between SARS-COV-2 infection and long-term depression symptoms among U.S. Veterans, this study evaluated the relationship between SARS-COV-2 infection and subsequent depression symptoms using data collected as part of a national survey nested within a larger matched cohort of Veterans who were and were not known to have SARS-COV-2 infections (see Method section). We hypothesized that SARS-COV-2 infection will be associated with increased depression symptoms compared with those uninfected at 18 months after initial infection. We also explored the role of past depression diagnosis to better assess the impact of new onset versus exacerbation of existing depression.

## METHODS

### Study Design, Setting, and Participants

We used national VHA electronic health records (EHR) to assemble 14 monthly cohorts (3/2020–4/2021) of SARS-COV-2 infected and contemporaneously uninfected Veterans matched on a broad range of baseline characteristics, including demographics, clinical history such as documented health conditions, geographical location, and healthcare utilization described in a previous protocol paper.^13^

We used a 25:1 matched design to ensure a sufficient number of comparators to recruit for survey completion. Match criteria were carefully chosen through consensus by VA SARS-COV-2 Outcomes Research Collaboratory (CORC) investigators and hypothesized to be risk factors or confounders for a broad range of pre-specified outcomes, including depression as per prior published work.^13,14^ To minimize missing EHR variables, we only included Veterans who had an assigned VA primary care team or at least one primary care clinic visit in the two years prior to the date of initial infection (or index date for matched uninfected). Clinical and demographics variables were extracted based on EHR data from any care encounter (e.g., ICD-10 codes from inpatient and outpatient visits) within two years prior to the index date. Index date was defined as the earliest date of SARS-COV-2 infection either from a VA-administered laboratory tests or patient self-report of a positive test from a test done outside the VA (Appendix Fig. 1). Matching was done with replacement such that Veterans without evidence of infection (in the EHR) prior to or during a given cohort month could be matched to more than one infected Veteran until they themselves became infected (Appendix Fig. 1).^13^

We invited a sample of 600 infected Veterans from the CORC cohort to participate in a telephone survey conducted approximately 18 months after their initial infection index date; uninfected comparators were surveyed based on a matching date. The infected Veterans were identified by sampling 100 Veterans who were infected in October, November, and December 2020 and February, March, and April of 2021. Veterans were not sampled in January of 2021 to ensure adequate resources for timely data collection. Sampling was stratified by US Census regions and hospitalization at time of infection to assure we captured broad geographic representation and illness spectrum. Survey interviews were conducted between May and December 2022. Once an infected member consented to participate in the survey, we recruited their 5 best-matched comparators to complete the same survey by telephone when possible; when more than one matched comparator responded, we analyzed the responses from the comparator who had been surveyed closest in time to the infected Veteran. Both infected Veterans and their matched comparators were asked to complete the same survey with no reference to their SARS-COV-2 history to minimize ascertainment and recall biases. To maximize survey completion rates, Veterans were given the option to complete the survey across multiple sessions and to return the survey by mail; we also used designated proxy respondents (i.e., primary caregivers) when Veterans were unavailable or needed assistance to complete the survey under an IRB-approved process. Survey participants were given a $10 incentive regardless of survey completion. Telephone surveys were administered by trained interviewers who entered responses directly into a secured REDCAP database with built-in validity and quality assurance checks.

### Outcomes Measurement

We used the Patient Health Questionnaire-9 (PHQ-9) to measure depression symptoms, which was administered as part of a larger survey evaluating the impact of SARS-COV-2 on physical and mental health functioning.^15–17^ The PHQ-9 is a multipurpose instrument used for screening, diagnosing, monitoring, and measuring the severity of depression in primary care and other medical settings. The instrument individually scores nine Diagnostic and Statistical Manual of Mental Disorders-5-TR depression diagnostic criteria on an ordinal scale based on their frequency ranging from 0 (not at all) to 3 (nearly every day) and provides a total score (range: 0 to 27).^18^ Higher PHQ-9 scores indicate greater depressive symptom severity and in validation studies have been shown to be associated with functional status, sick days, and healthcare utilization.^15–17^

### Primary Exposure

SARS-COV-2 infection was our primary exposure of interest. We used the VHA’s COVID-19 Shared Data Resource, a collection of SARS-COV-2-related data resources to expedite research across the VHA, to identify Veterans with documentation of SARS-COV-2 infection (laboratory tests or self-report to VA) in each month during the study period.^19^ Veterans without evidence of infection during and prior to each given month were eligible to serve as uninfected matched comparators.

### Statistical Methods

#### Outcome variable specification

We analyzed total PHQ-9 scores as both a continuous measure and binary indicator using the standard score ≥ 10 as indicating high risk for major depression based on established clinical cut-offs.^17^ Furthermore, we constructed PHQ-9 ‘psychological’ (e.g., negative self-worth) and ‘physical’ (e.g., appetite changes) subdomain scores using an approach described by Fleethart et al.^20^ However, we included Item 9 in our PHQ-9 psychological measure as we did not stratify our analyses on suicidal ideation. We analyzed these domain-specific scores as continuous outcomes (range: 0–12 for psychological; range: 0–15 for physical).

When the PHQ-9 was partially completed, we imputed responses for the missing items using the average of the observed responses.^16,21^ For example, if the average score of 8 of 9 items was 2.5, we imputed 2.5 for the missing item and took the sum of all observed and imputed values as the total score to decrease bias due to missingness. This imputation was only done when missing 3 or fewer (≤ 33%) items. For sub-domain scores, weighted scores were imputed only when 1 item was missing in each respective sub-domain. Overall, PHQ-9 items were rarely missing (2%). We found no major differences in demographics and clinical characteristics based on measure completion.

#### Survey Weights

We constructed analytic weights to approximate a representative sample of our larger EHR-based matched cohorts. First, sampling weights were applied to all cohort members invited to participate in the survey proportional to their probability of being sampled. Second, survey-level non-response weights were created by estimating the probability of non-response among all invited to participate in the survey using a logistic regression model with age, sex-assigned-at-birth, race, and Gagne index (a measure of comorbidity disease burden) scores as predictors including additional matching variables selected using a lasso procedure.^22^ These variables were selected to model nonresponse, because we hypothesized they were predictive of a participant’s propensity to respond. For example, patients with a higher Gagne index who experienced more physical health challenges may be less likely to participate. The final analytic weight was the product of sampling weights and non-response weights. All reported analyses and results are weighted.

#### Descriptive Statistics

We used descriptive statistics to compare baseline characteristics of infected and uninfected Veterans and used standardized mean differences (SMDs) to assess group differences (Table 1). We used histograms and density plots to compare the distribution of total PHQ-9 and subdomain scores between infected and uninfected Veterans. To better understand potential risk factors for depression, we summarized baseline characteristics among those who screened positive for depression (PHQ-9 ≥ 10) compared to those who did not.

**Table 1:** Descriptive Characteristics of Primary Study Sample

	Overall *N* = 372	Case *N* = 186	Comparator *N* = 186	SMD*
Sex				0.000
Female	44 (9.5%)	22 (9.5%)	22 (9.5%)	
Male	326 (90.4%)	163 (90.4%)	163 (90.4%)	
Missing	2 (0.1%)	1 (0.1%)	1 (0.1%)	
Age (years)				-0.057
Median (IQR)	62.0 (51.00, 72.0)	60.0 (53.00, 71.0)	64.0 (51.00, 72.0)	
Mean (SD)	60.21 (13.9)	59.82 (13.5)	60.60 (14.2)	
Race				0.137
Black	93 (27.3%)	46 (30.3%)	47 (24.3%)	
White	244 (66.9%)	128 (64.5%)	116 (69.4%)	
Another Identity^a^	35 (5.8%)	12 (5.3%)	23 (6.3%)	
Ethnicity				0.233
Latine	31 (5.8%)	12 (3.2%)	19 (8.3%)	
Not Latine	328 (92.1%)	167 (94.0%)	161 (90.2%)	
Missing	13 (2.1%)	7 (2.8%)	6 (1.5%)	
Urban Residence	254 (67.9%)	130 (73.1%)	124 (62.7%)	0.224
Immunosuppressed	30 (11.1%)	15 (11.1%)	15 (11.1%)	0.000
Coronary Heart Disease	81 (20.7%)	39 (18.6%)	42 (22.9%)	0.104
Cancer	66 (19.1%)	32 (19.7%)	34 (18.5%)	0.030
Chronic Kidney Disease	76 (24.4%)	36 (25.7%)	40 (23.0%)	0.063
Congestive Heart Failure	29 (7.3%)	15 (7.7%)	14 (6.9%)	0.029
Pulmonary Disease	69 (18.5%)	33 (17.1%)	36 (19.9%)	0.071
Dementia	8 (1.3%)	2 (0.6%)	6 (1.9%)	0.110
Diabetes	128 (36.1%)	69 (35.8%)	59 (36.4%)	0.013
Hypertension	234 (64.5%)	116 (61.6%)	118 (67.4%)	0.121
Liver Disease	26 (7.4%)	14 (9.5%)	12 (5.4%)	0.156
Stroke/Cerebrovascular	10 (2.8%)	6 (1.3%)	4 (4.2%)	0.176
Substance Use Disorder	44 (10.1%)	23 (12.2%)	21 (8.0%)	0.140
Anxiety	85 (22.3%)	51 (25.9%)	34 (18.7%)	0.174
Bipolar	15 (2.8%)	6 (2.0%)	9 (3.6%)	0.096
Major Depression	128 (32.3%)	67 (33.6%)	61 (31.1%)	0.054
PTSD	94 (22.3%)	43 (21.1%)	51 (23.5%)	0.058
Schizophrenia	6 (0.8%)	0 (0.0%)	6 (1.6%)	*
Smoking Status				0.212
Current	41 (10.8%)	17 (9.3%)	24 (12.3%)	
Former	151 (45.0%)	78 (47.1%)	73 (42.8%)	
Never	163 (38.9%)	83 (40.3%)	80 (37.5%)	
Missing	17 (5.3%)	8 (3.3%)	9 (7.4%)	
No. CDC High Risk Conditions				-0.026
Median (IQR)	2.0 (1.00, 3.0)	2.0 (1.00, 3.0)	2.0 (1.00, 3.0)	
Mean (SD)	2.12 (1.7)	2.10 (1.6)	2.15 (1.8)	
No. CDC High Risk Mental Health Conditions				0.040
0	185 (53.3%)	89 (51.7%)	96 (54.8%)	
1	85 (23.0%)	45 (23.0%)	40 (22.9%)	
2	69 (15.2%)	35 (16.5%)	34 (14.0%)	
3	27 (7.1%)	16 (8.4%)	11 (5.7%)	
4	6 (1.5%)	1 (0.3%)	5 (2.6%)	
No. Inpatient Admissions				-0.094
Median (IQR)	0.0 (0.00, 0.0)	0.0 (0.00, 0.0)	0.0 (0.00, 0.0)	
Mean (SD)	0.39 (1.1)	0.34 (1.0)	0.44 (1.2)	
No. Primary Care Visits				-0.019
Median (IQR)	7.0 (3.00, 14.0)	7.0 (3.00, 12.0)	6.6 (3.00, 14.0)	
Mean (SD)	10.62 (11.2)	10.51 (10.7)	10.72 (11.6)	
No. Specialty Care Visits				-0.064
Median (IQR)	11.0 (5.00, 23.0)	11.0 (5.00, 20.2)	11.0 (5.00, 24.4)	
Mean (SD)	16.44 (17.3)	15.88 (15.5)	17.00 (19.0)	
No. Mental Health Visits				0.272
Median (IQR)	0.0 (0.00, 4.0)	0.0 (0.00, 6.6)	0.0 (0.00, 2.0)	
Mean (SD)	5.62 (14.2)	7.53 (17.5)	3.72 (9.5)	
Body Max Index (kg/m2)				-0.220
Median (IQR)	30.6 (27.01, 34.6)	30.1 (26.99, 33.6)	31.3 (27.20, 36.1)	
Mean (SD)	31.52 (6.5)	30.81 (5.5)	32.23 (7.3)	
Gagne Index				0.097
Median (IQR)	0.0 (0.00, 2.0)	1.0 (0.00, 2.0)	0.0 (0.00, 2.0)	
Mean (SD)	1.18 (2.0)	1.28 (1.8)	1.08 (2.2)	
Nosos Score				-0.007
Median (IQR)	0.9 (0.63, 1.4)	0.9 (0.58, 1.4)	0.9 (0.68, 1.3)	
Mean (SD)	1.21 (0.9)	1.21 (0.9)	1.22 (0.9)	
CAN Score				0.065
Median (IQR)	65.0 (35.00, 80.0)	65.0 (35.00, 84.1)	60.0 (35.00, 80.0)	
Mean (SD)	56.69 (28.1)	57.61 (29.0)	55.78 (27.3)	
Distance to VA Medical Center (miles)				-0.092
Median (IQR)	23.4 (10.68, 47.5)	21.3 (8.28, 47.5)	25.3 (14.53, 47.1)	
Mean (SD)	35.60 (35.9)	33.97 (37.5)	37.24 (34.2)	

SMD = Standardized Mean Difference; CAN = VA Care Assessment Need

* SMD for schizophrenia not estimated because no diagnoses among cases

^a^Those in the “Another Identity” group consisted of identities with small sample sizes in our study including those with unknown racial identity, Native American, Asian, Native Hawaiian or Pacific Islander, and more than one racial ethnic identity

Ns are unweighted. Descriptive statistics are survey weighted. The primary study sample excludes the matched pairs where the comparator became infected with SARS-COV-2 between their matched index date and survey completion date

#### Primary Analyses

For our continuous measures (overall PHQ-9 and subdomain scores), we estimated a mean difference in scores between those infected with SARS-COV-2 and comparators using weighted generalized estimating equations (GEEs), specified with a Gaussian family with identify link and exchangeable correlation structure, and estimated robust sandwich-type standard errors (clustered on match group) to account for the matched design.^23^ For the binary outcome of positive depression screening, we used weighted GEEs to estimate the relative risk (Poisson family with log link) and risk difference (binomial family with identity link), again with exchangeable correlation structure and robust standard errors. To account for potential residual confounding post-matching, all model effect estimates were adjusted by including covariates with SMDs > 0.1 identified in descriptive analyses. We used diagnostic plots to assess linear model assumptions which were found to be met adequately.

Our primary analysis estimates a ‘per-protocol’ effect by excluding matched pairs where the matched, uninfected respondent became infected (identified based on the process described above) in the18-months between their match index date and the survey administration date. For all analyses, the significance level was set at *p* < 0.05. We provide estimates of Cohen’s *d* for primary outcomes to facilitate interpretation of findings.^24^

#### Sensitivity Analyses

We estimated an analogous ‘intent-to-treat’ effect including all pairs as a secondary analysis. A post-hoc subgroup analysis was done to estimate the effect of infection on 18-month PHQ-9 scores among those with and without a history of diagnosed depression based on the presence of a depression diagnosis in administrative data within the past two years. We used the same unadjusted and adjusted GEEs used in the per-protocol analysis and included an interaction term between infection status and prior depression diagnosis to assess effect modification by depression history. All analyses were conducted in R (version 4.1.2).

### Ethics/IRB Statement

The study was reviewed and approved by the Institutional Review Boards of the Ann Arbor, Durham, Palo Alto, Portland, and Puget Sound VHA Medical Centers. All participants provided informed consent to participate in the study.

## RESULTS

### Analytic Sample

Among 600 SARS-COV-2 infected cohort members sampled, 548 (91%) were still alive and residing in the U.S. at the time of survey administration, and 235 (43%) consented and completed surveys. Among the 235 infected Veteran survey completers, we received surveys from 194 (83%) of their matched comparators for a total of 388 complete surveys (194 in each match group). Eight (4%) matched comparators became infected with SARS-COV-2 prior to survey administration, so the per protocol sample excluding the matched sets with an infected comparator included 372 Veterans (186 per group). Among all survey respondents, 9 (2%) left at least one PHQ-9 prompt unanswered (6 infected, 3 comparators), and 3 who did not complete any PHQ-9 prompt (1 case, 2 comparators) were excluded from analysis. After imputing final scores for Veterans with at least one PHQ-9 item completed, 4 Veterans were missing PHQ-9 outcomes and excluded from analyses. We found few differences in baseline characteristics between those with and without complete PHQ-9 responses. Those missing PHQ-9 outcomes were all male, 3 (75%) had prior major depression diagnoses, and had descriptively more prior mental health care visits.

### Primary Results – Descriptive Statistics

Veterans in the per protocol sample (*n* = 372) had a median age of 62 years (IQR: 51 -72), were predominantly male (90%), and nearly all non-Latine (92%). There were some descriptive differences (SMD > 0.10) between COVID-infected vs. uninfected participants on several characteristics, including body mass index, pre-existing conditions (e.g., anxiety), race, and ethnicity (Table 1). Mean PHQ-9 score was 8.0 (SD: 6.5) in the overall sample and was higher among Veterans with SARS-COV-2 (M: 9.2, SD: 6.8) compared with matched comparators (M: 6.8, SD: 6.0; Table 2). For all individual PHQ-9 items, a greater proportion of Veterans with SARS-COV-2 reported experiencing symptoms ‘more than half the days’ or ‘nearly every day’ than comparators (Appendix Fig. 2). For example, over a third of Veterans with SARS-COV-2 reported feeling down, depressed, or hopeless at least more than half the days compared with 16% of comparators.

**Table 2:** Summary of PHQ-9 Outcomes in the Primary Study Sample Overall and by SARS-COV-2 Status

	Overall *N* = 372	Case *N* = 186	Comparator *N* = 186
Total PHQ9 Score			
Median (IQR)	7.0 (2.00, 12.0)	9.0 (3.00, 14.1)	5.0 (2.00, 10.0)
Mean (SD)	7.98 (6.5)	9.18 (6.8)	6.78 (6.0)
Major Depression Screen			
Lower Risk (< 10)	233 (62.2%)	102 (50.7%)	131 (73.7%)
Higher Risk (10–27)	135 (37.6%)	82 (49.0%)	53 (26.1%)
Missing	4 (0.2%)	2 (0.3%)	2 (0.2%)
PHQ9 Depression Rating			
Minimal (0–4)	142 (39.9%)	63 (33.4%)	79 (46.4%)
Mild (5–9)	91 (22.3%)	39 (17.3%)	52 (27.3%)
Moderate (10–14)	63 (18.5%)	37 (24.2%)	26 (12.8%)
Moderately severe (15–19)	49 (14.0%)	30 (18.2%)	19 (9.8%)
Severe (20–27)	23 (5.1%)	15 (6.6%)	8 (3.5%)
Missing	4 (0.2%)	2 (0.3%)	2 (0.2%)
PHQ Psychological Domain			
Median (IQR)	2.0 (0.00, 5.0)	2.0 (0.00, 5.0)	1.0 (0.00, 3.0)
Mean (SD)	2.56 (2.8)	3.16 (2.9)	1.96 (2.6)
PHQ Physical Domain			
Median (IQR)	5.0 (2.00, 9.0)	6.0 (2.16, 9.0)	4.0 (1.00, 7.0)
Mean (SD)	5.41 (4.2)	6.01 (4.3)	4.82 (4.0)

Ns are unweighted. Descriptive statistics are survey weighted

### Primary Results – Main Outcome Analyses

After adjusting for imbalanced confounders in the models, those infected had higher mean overall PHQ-9 scores (mean difference: 2.24, 95% CI: 0.70, 3.78; *d* = 0.38) and psychological subdomain scores (mean difference: 1.23, 95% CI: 0.59, 1.88; *d* = 0.44) than comparators (Fig. 3). Differences in the physical subdomain score were not statistically significant (mean difference: 0.99, 95% CI: -0.03, 2.02; *d* = 0.29). There was a significantly higher absolute 18-month risk of screening positive for major depression among Veterans with SARS-COV-2 (risk difference: + 22 percentage points, 95% CI: 10, 33) and higher relative risk (1.89, 95% CI: 1.30, 2.75).

### Sensitivity Analyses

The intent-to-treat analysis showed similar results, except that the mean difference in the physical subdomain was greater among infected Veterans and statistically significant (mean difference: 1.08, 95% CI: 0.10, 2.07; Appendix Fig. 3). In our post-hoc subgroup analysis, comparing outcomes among those with and without a prior depression diagnosis, none of the tests for interaction with history of depression reached statistical significance (all *p* > 0.05; Appendix Fig. 3).

## DISCUSSION

As hypothesized, those with SARS-COV-2 infection had higher mean depression scores than those who had not been infected with a small effect at 18 months post-index date. In contrast to prior research by Na and colleagues, our study showed a significantly higher prevalence of positive depression screening (49% versus 11.8%).^11^ As their paper used an abbreviated, four-item depression screening instrument, it is possible that the prevalence in their sample was underestimated due to exclusion of other depression symptoms (e.g., suicidal ideation, negative self-worth) or a shorter follow-up time frame (one year versus 18 months).^11^

When considering depression subdomains, psychological symptoms of depression were elevated among those with SARS-COV-2 infection showing a small-to-moderate difference in effect, but physical symptoms of depression were not. Existing models of adjustment to illness posit that acute (e.g., burden of treatment, receiving a diagnosis) and ongoing (e.g., impacts on social networks) illness stressors impact cognitive, emotional, and behavioral reactions that contribute to overall health and well-being.^25^ Consequently, it is unsurprising that SARS-CoV-2 infection may precipitate long-term, sustained elevations in depression symptoms. Although many studies on ongoing illness stressors associated with COVID-19 have focused on physical symptoms, our findings suggest that additional intervention focused specifically on psychological symptoms of depression is needed for improving health outcomes long after initial infection.^26,27^

In exploring the potential interaction of history of depression and SARS-CoV-2, we found no statically significant interaction effects between history of depression and SARS-CoV-2 infection. While it is possible that the association of SARS-COV-2 infection with depression symptoms is similar regardless of prior depression history, our study may be underpowered to detect this interaction. In light of our findings, clinicians may wish to consider evaluating post-COVID depression symptoms regardless of depression history to ensure adequate intervention and support for those infected with SARS-COV-2.

### Limitations

Our study has several notable strengths including the robust matching design based on an emulated trials framework and statistical weighting to account for potential biases in the sample due to mortality or later SARS-CoV-2 infection. In addition, we utilized measurement of long-term depression symptoms at a later outcome timepoint than past studies and use of patient-reported measures. Despite the strengths of our study design, there are several limitations. As we only measured depression symptoms at one time point (18-months post-infection), we were unable to determine how depression symptoms may have changed both prior to or directly following infection as well as whether such symptoms are secondary to Long COVID. It is possible some SARS-CoV-2 infections were not captured in our data due to unreported home testing. Future research assessing additional measurement time points may better characterize trajectories of depression symptoms not only 18 months after SARS-CoV-2 infection but also over shorter and longer timeframes. Finally, our Veteran population tends to have more comorbidities than non-Veterans (e.g., Veterans have elevated rates of kidney disease and diabetes.) which may limit generalizability.^28^

### Implications

Among participants in a survey study specifically designed to account for confounding biases, we observed substantially elevated prevalence of depression symptoms 18 months after initial SARS-CoV-2 infection. Although the overall difference was in the small effect range, clinicians should consider incorporating standardized assessment of depression symptoms among those infected with SARS-CoV-2 to mitigate mental health morbidity in light of heterogeneity of symptoms across those infected. Notably, differences were more pronounced in the psychological symptoms subscale than the physical symptoms subscale. Future research evaluating depression symptoms should consider exploring these subscales in addition to total scores to better disentangle SARS-CoV-2 physical impacts (e.g., sleep disruption) from depressed mood.

## Conclusion

SARS-CoV-2 infection was associated with increased depression symptoms among Veterans 18-months post-infection. Routine evaluation of depression symptoms following SARS-CoV-2 infection is important to facilitate adequate assessment and treatment.

### Supplementary Information

Below is the link to the electronic supplementary material.

Supplementary file1 (DOCX 235 KB)
