# Supplementary material for: Impact of SARS-CoV-2 Infection on Long-Term Depression Symptoms among Veterans
Source: J Gen Intern Med. 2024 Apr 16;39(8):1310–6. doi: 10.1007/s11606-024-08630-z (PMC11169300; doi:10.1007/s11606-024-08630-z)

**Figure. Unadjusted and adjusted effect estimates for all primary outcomes (survey weighted). Continuous PHQ-9 scores are presented as mean differences. Results for screening positive for major depression (PHQ-9 score ≥10) are presented on both the absolute risk difference scale and relative risk ratio scale.**


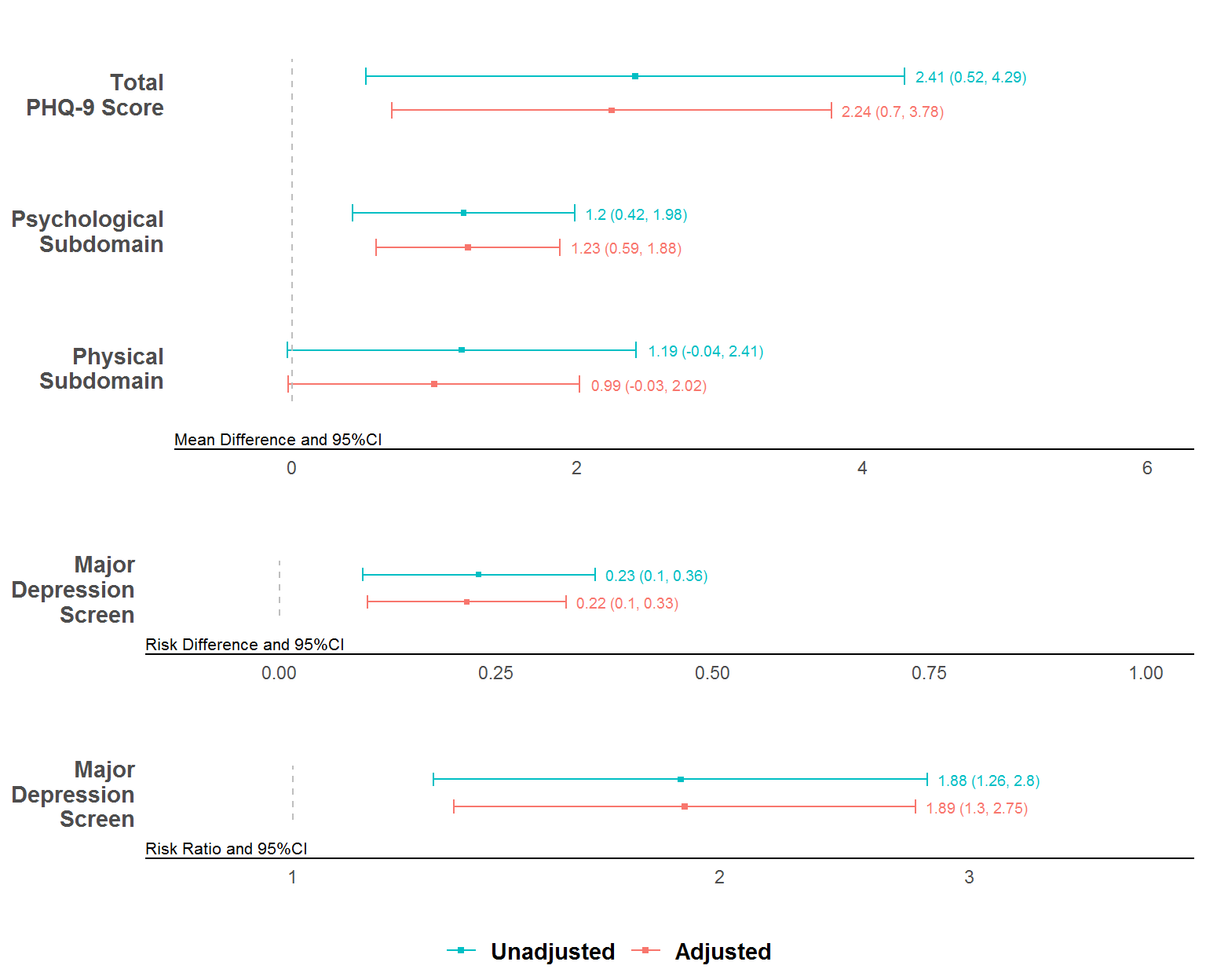


**Appendix Figure 1. Study Flow Diagram
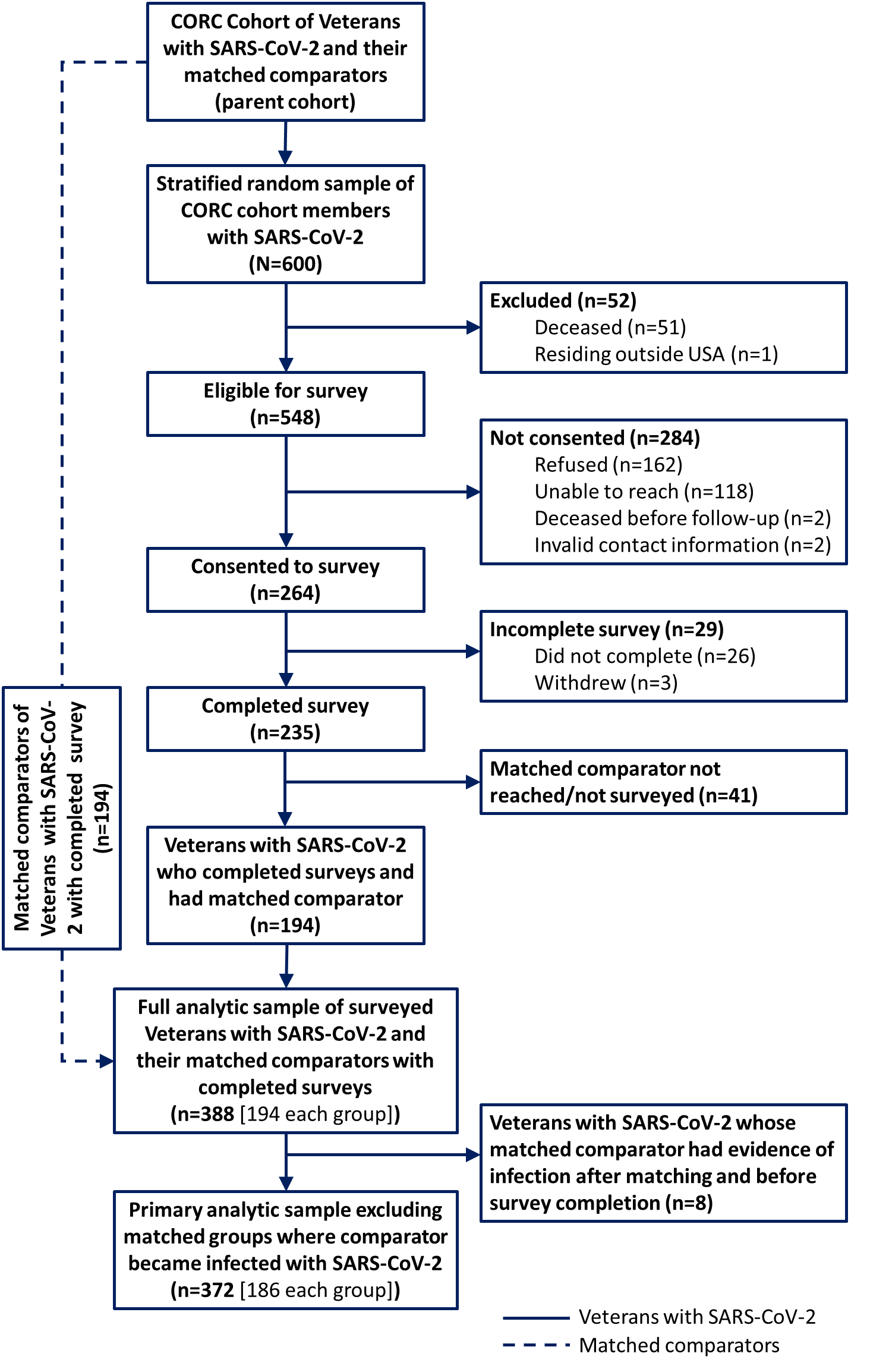
**

**Appendix Figure 2. Item-level Frequencies Comparing Those With and Without SARS-CoV-2 Infection**


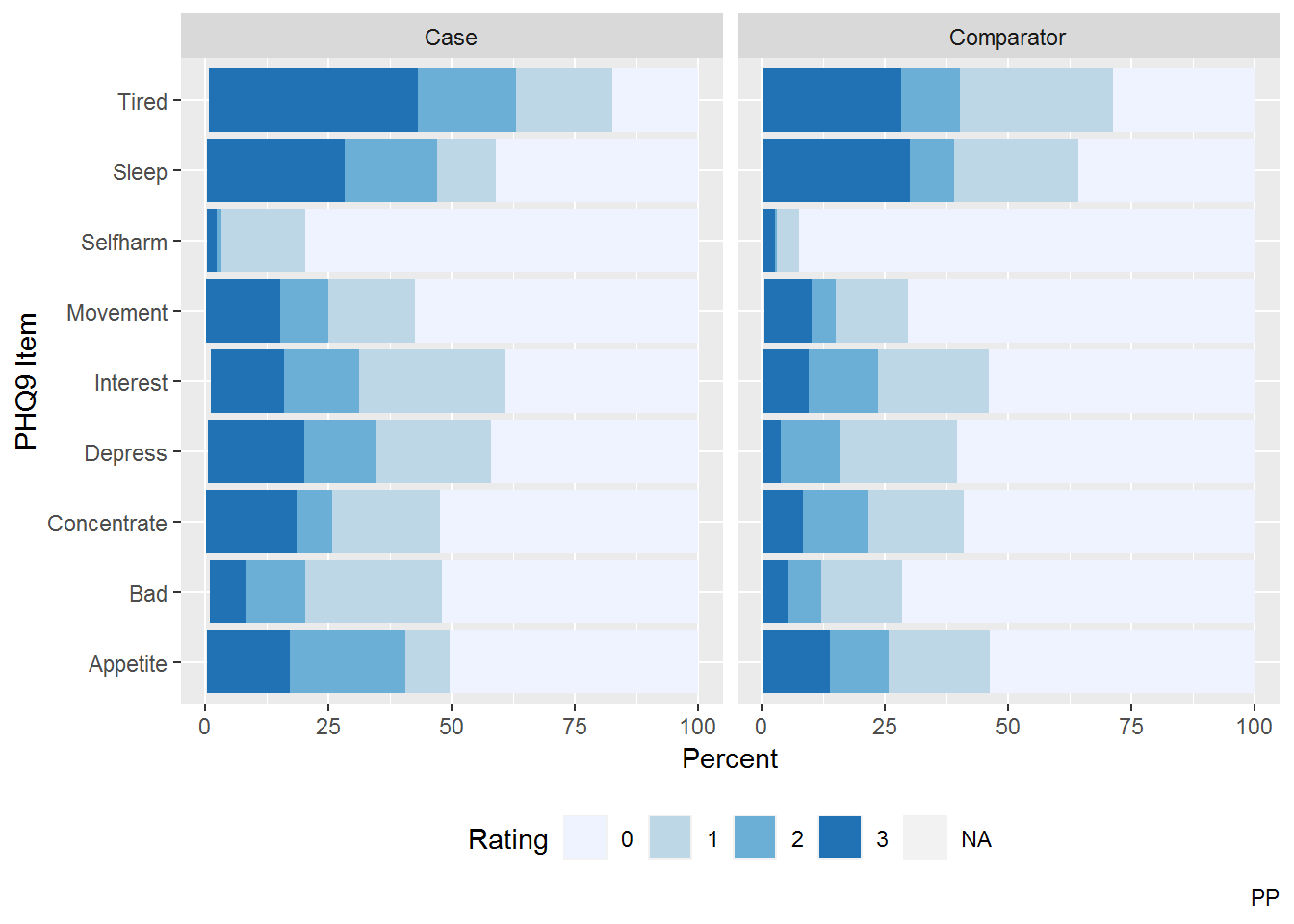


**Appendix Figure 3. Intent-to-Treat Sensitivity Analysis**


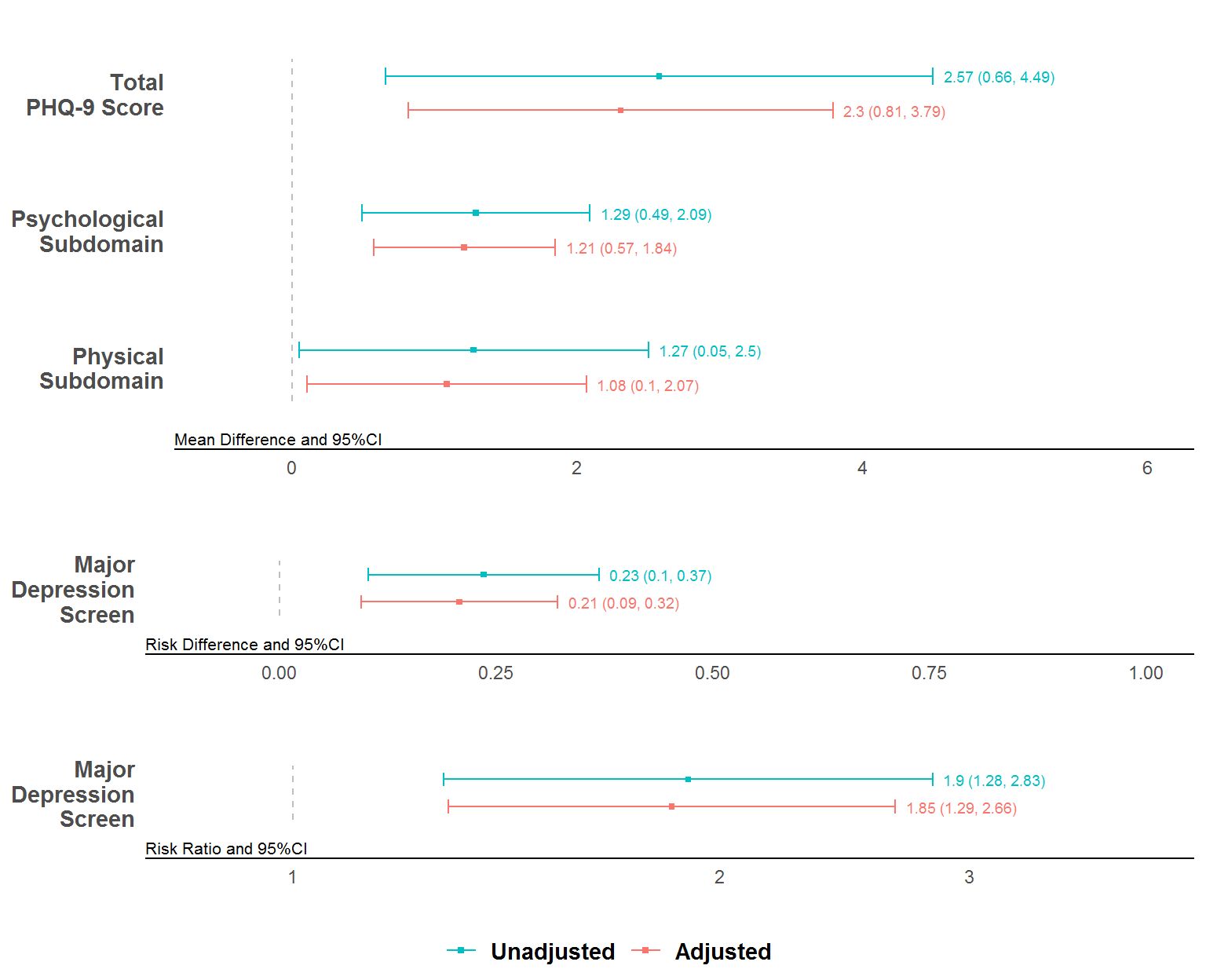

Supplement: Supplementary file 1 — Supplementary file1 (DOCX 235 KB) [file 11606_2024_8630_MOESM1_ESM.docx]
